# Hyperoside Alleviates Myocardial Fibrosis by Reducing Oxidative Stress via the GATA4/HIF-1α Pathway in Mice

**DOI:** 10.3390/ph19050755

**Published:** 2026-05-12

**Authors:** Xiaotong Dou, Haofang Wan, Tianxue Chen, Huifen Zhou, Li Yu, Haitong Wan

**Affiliations:** 1 School of Basic Medical Sciences, Zhejiang Chinese Medical University, Hangzhou 310053, China; 2 Zhejiang Key Laboratory of Chinese Medicine for Cardiovascular and Cerebrovascular Disease, Hangzhou 310053, China; 3 College of Chinese Medicine for Cardiovascular-Cranial Disease, Zhejiang Chinese Medical University, Hangzhou 310053, China; 4 Academy of Chinese Medical Sciences, Zhejiang Chinese Medical University, Hangzhou 310053, China

**Keywords:** hyperoside, isoproterenol induced myocardial fibrosis, oxidative stress, GATA4/HIF-1α pathway, mice, molecular docking

## Abstract

**Background/Objective**: Myocardial fibrosis (MF) is a prevalent pathological endpoint in various heart diseases, characterized by extracellular matrix (ECM) dysregulation and oxidative stress. Hyperoside (Hyp) plays a role in regulating cardiac oxidative stress and fibrosis. This study aimed to elucidate whether Hyp regulates isoproterenol (ISO)-induced MF in mice by modulating the GATA4/HIF-1α signaling pathway and reducing oxidative stress. **Methods**: The binding affinity of Hyp to GATA4 and HIF-1α was assessed through molecular docking and dynamics simulation. The MF model of mice was established by subcutaneous injection of ISO. Cardiac function was measured by echocardiography. Myocardial injury and collagen deposition were examined using H&E and Sirius red staining. Levels of fibrosis markers, oxidative stress indicators, and GATA4/HIF-1α pathway indicators in serum and heart tissue were quantified by ELISA, Western blot, RT-qPCR and flow cytometry. The distribution of myocardial marker proteins was visualized by immunofluorescence and immunohistochemistry. **Results**: Molecular docking revealed high binding affinity of Hyp to GATA4 and HIF-1α (binding energies < −5.0 kcal·mol^−1^), and dynamics simulation showed that the complex’s structure remained stable over 100 nanoseconds (RMSD < 0.1 nm). High-dose Hyp (36 mg/kg) significantly improved cardiac function, myocardial injury, collagen deposition, and inflammatory infiltration in MF mice. Molecularly, Hyp effectively reduces oxidative stress and fibrosis through upregulating GATA4 and downregulating HIF-1α. **Conclusions**: Hyp suppresses oxidative stress by activating the GATA4/HIF-1α pathway, presenting a promising therapeutic target for the treatment of MF.

## 1. Introduction

Cardiovascular diseases have become a major threat to public health due to their high mortality and morbidity [1]. Myocardial fibrosis (MF) is not only a key link in cardiac remodeling, but also an important pathological basis for the progression of various heart diseases [2]. The abnormal repair response triggered by long-term ischemia, hypoxia or inflammation in the heart leads to enhanced oxidative stress and imbalance of the synthesis and degradation of extracellular matrix (ECM) [3], and causes excessive deposition of collagen fibers and spatial structure remodeling, eventually leading to increased myocardial stiffness, limited diastolic function, and even deterioration of overall cardiac function [4,5]. However, the therapeutic strategies of MF still face many difficulties due to its complex pathogenic mechanisms. Therefore, in-depth exploration of its molecular mechanism and development of new treatment strategies have become an important research direction in the current cardiovascular field. In recent years, Hyperoside (Hyp) has gradually become a research hotspot because of its multi-target regulation mechanism to improve myocardial injury, which provides a new research idea for the intervention of MF.

Hyp, a flavonoid glycoside organic compound commonly found in plants [6], has significant physiological activity, including neuroprotective, anti-inflammatory, anti-cancer, antioxidant, and cardiovascular protective effects [7,8,9,10]. Hyp can regulate autophagy by inhibiting the NLRP1 inflammatory pathway and alleviate ventricular remodeling in mice with myocardial infarction [11], and reduce doxorubicin-induced cardiotoxicity by blocking the NOX/ROS/NLRP3 inflammasome pathway [12]. It also inhibits angiotensin II-induced cardiomyocyte hypertrophy and trastuzumab-induced cardiomyocyte apoptosis by activating the PI3K/Akt signaling pathway [13,14]. These studies suggest that Hyp has significant multi-dimensional potential in cardiac protection related to oxidative stress, inflammatory response, energy metabolism and apoptosis. However, the role of Hyp in MF and its underlying mechanism are not yet clear.

GATA Binding Protein 4 (GATA4) is a key transcription factor that regulates heart development, differentiation and adult heart homeostasis [15]. Recent evidence indicates that GATA4 overexpression can reverse cardiac fibrosis and diastolic impairment in heart failure with preserved ejection fraction (HFpEF) [16]. Hypoxia-Inducible Factor-1α (HIF-1α) is a core member of the HIF family [17], and its excessive activation promotes oxidative stress, inflammation and fibrosis. There is a close functional synergy and regulatory interaction between GATA4 and HIF-1α in the heart, which together constitute the key pathway of myocardial protection. Studies indicated that latifolin promotes the repair of bone marrow mesenchymal stem cells to ischemic heart disease by upregulating GATA4 and downregulating HIF-1α [18]. In addition, GATA4 and HIF-1α are indirectly associated through Notch signaling to promote early cardiac stem cells differentiation [19]. However, it is not clear whether Hyp alleviates MF through the GATA4/HIF-1α pathway.

Accordingly, the present study mainly elucidates whether the mechanism of Hyp against MF is related to regulating oxidative stress via the GATA4/HIF-1α signaling pathway. The interaction mode between Hyp and GATA4/HIF-1α was analyzed through molecular docking and molecular dynamics simulation. The anti-fibrosis effect of Hyp on this pathway was verified by combining the mouse MF model induced by isoproterenol (ISO) and overexpression of GATA4, systematically exploring the key signaling pathway of Hyp. The findings are expected to establish a foundational framework and furnish essential experimental evidence to advance the development of Hyp as a therapeutic agent for MF.

## 2. Results

### 2.1. Molecular Docking and Dynamic Simulation Analysis of Hyp on Key Targets of GATA4/HIF-1α Pathway

**Figure 1:**
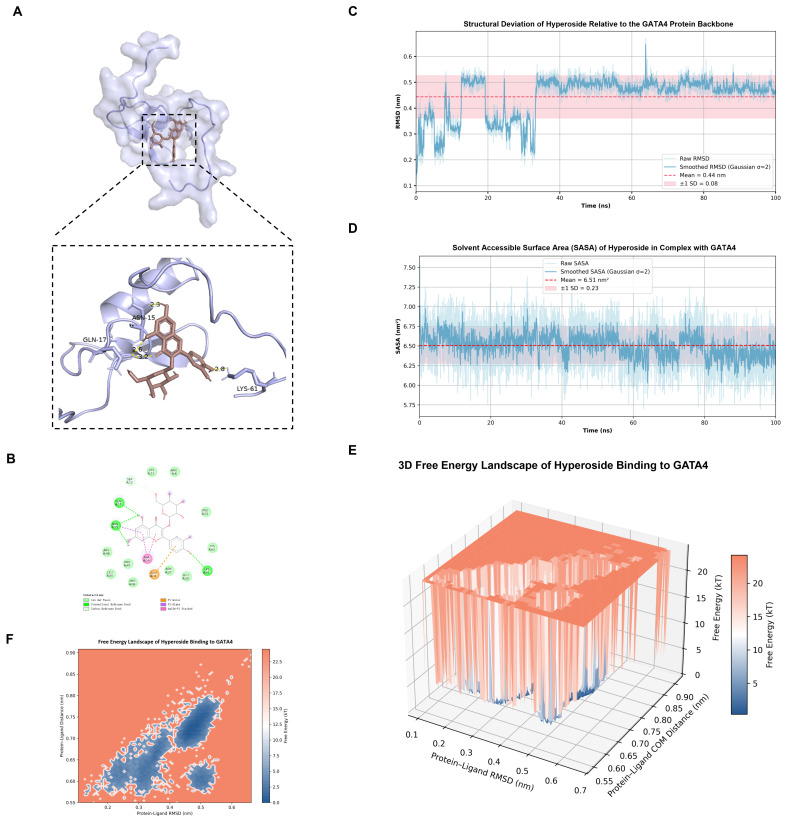
Molecular docking and molecular dynamics simulation of Hyp-GATA4 complex. (**A**) Three-dimensional docking conformation of Hyp-GATA4 complex. (**B**) Two-dimensional interaction diagram of Hyp-GATA4 complex. (**C**) RMSD distribution of Hyp relative to GATA4 skeleton. The dark blue line represents the RMSD trajectory smoothed by Gaussian filtering (σ = 2), the red dotted line represents the average RMSD, and the light red region shows a fluctuation range of ±0.01 nm. (**D**) Solvent accessible surface area (SASA) distribution map of Hyp in GATA4 complex. The meanings of dark blue lines, red dashed lines and light red areas are the same as those in (**C**). (**E**) Three-dimensional free energy landscape of Hyp-GATA4 complex. (**F**) Two-dimensional free energy surface of Hyp-GATA4 complex.

**Figure 2:**
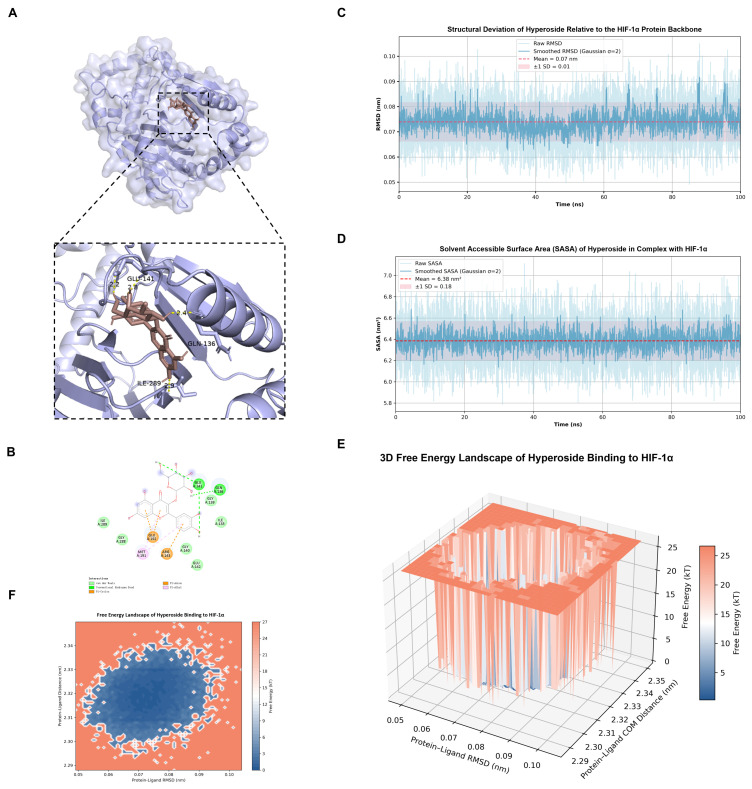
Molecular docking and molecular dynamics simulation of Hyp-HIF-1α complex. (**A**) Three-dimensional docking conformation of Hyp-HIF-1α complex. (**B**) Two-dimensional interaction diagram of Hyp-HIF-1α complex. (**C**) RMSD distribution of Hyp relative to HIF-1α skeleton. The dark blue line represents the RMSD trajectory smoothed by Gaussian filtering (σ = 2), the red dotted line represents the average RMSD, and the light red region shows a fluctuation range of ±0.01 nm. (**D**) Solvent accessible surface area (SASA) distribution map of Hyp in HIF-1α complex. The meanings of dark blue lines, red dashed lines and light red areas are the same as those in (**C**). (**E**) Three-dimensional free energy landscape of Hyp-HIF-1α complex. (**F**) Two-dimensional free energy surface of Hyp-HIF-1α complex.

Molecular docking analysis was performed to assess the potential interaction between Hyp and GATA4, HIF-1α. Docking results indicated that Hyp bound to GATA4 and HIF-1α, exhibiting binding energies < −5.0 kcal·mol^−1^, which was consistent with the binding potential and had good affinity (Figure 1A,B and Figure 2A,B). Table 1 summarizes the specific binding energies.

Through in-depth analysis of the 100 ns simulation trajectory, the structural stability and energy surface characteristics of Hyp binding to HIF-1α and GATA4 proteins were evaluated. In the Hyp-HIF-1α complex, the system exhibits extremely high structural rigidity, and the RMSD trajectory diagram (Figure 1C) shows that the fluctuation is very small, with an average value of only 0.07 ± 0.01 nm, indicating that the binding conformation remains locked throughout the simulation process, and the structural stability is excellent. The SASA (Figure 1D) of the complex remains highly constant, with an average of about 6.38 ± 0.18 nm^2^, suggesting that the exposed conformation of the ligand in the binding pocket is extremely stable. Hydrogen bond analysis indicated that the system maintained 1–2 high-frequency hydrogen bonds, which constituted a stable anchoring effect. The corresponding free energy landscape (FEL) displays a deep and narrow single energy valley, and the system is evidently concentrated in the lowest energy region (blue region, centered about RMSD 0.07 nm), and the surrounding energy barrier is more than 25 kT, which further confirms that its conformation is extremely limited and the binding mode is extremely robust (Figure 1E,F).

In contrast, the Hyp-GATA4 complex exhibits a more dynamic conformational optimization process. As depicted in the RMSD trajectory (Figure 2C), the system undergoes a significant conformational adjustment during the initial simulation stage (0–35 ns), with the RMSD fluctuating between 0.2 and 0.5 nm before gradually reaching a dynamic equilibrium. The average value of the whole trajectory is 0.44 ± 0.08 nm. After 35 ns, the RMSD curve tended to be stable without obvious drift, which verified the reliability of the complex structure and Hyp chimerism. SASA Analysis (Figure 2D) showed that the mean value was 6.51 ± 0.23 nm^2^, and the stability of the fluctuation reflected that the ligand had reached the best encapsulation state in the binding pocket. Unlike the HIF-1α system, the GATA complex is stable through a richer hydrogen bond network, and the number of hydrogen bonds fluctuates frequently between 2 and 5, with a peak of up to 7. FEL analysis (Figure 2E,F) shows that the system contains a main energy deep well (RMSD~0.5 nm) and several steady-state troughs, indicating that Hyp finally converges to a low-energy, stable state through multi-level conformational search at the GATA4 site, and its characteristics are consistent with the results of structural fluctuations. In general, Hyp demonstrated significant binding affinity and conformational stability in both target systems by using the multi-point synergy of its aromatic skeleton, which constituted the structural basis of its potential biological activity.

### 2.2. Hyp Improved Cardiac Function in MF Mice

**Figure 3:**
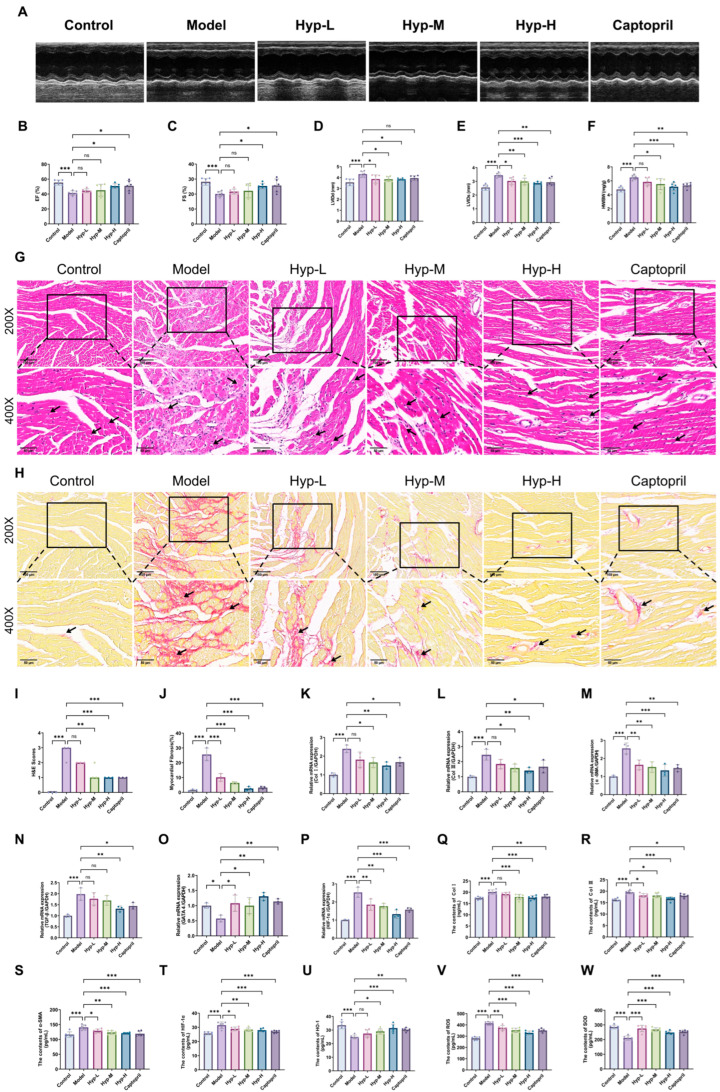
Effects of different doses of Hyp on MF in mice. (**A**) Representative diagram of echocardiography in mice. (**B**–**E**) EF, FS, LVIDd and LVIDs were measured by echocardiography in mice ($\bar{x}$ ± s, *n* = 6). (**F**) Cardiac index ($\bar{x}$ ± s, *n* = 6). (**G**,**I**) H&E staining representative figure and H&E score of mouse heart tissue (Data are presented as median (IQR), *n* = 3). Black arrows indicate inflammatory cell infiltration (200×: 100 μm; 400×: 50 μm). (**H**,**J**) Sirius Red staining of mouse heart tissue and myocardial collagen volume fraction ($\bar{x}$ ± s, *n* = 3). Black arrows indicate collagen deposition (200×: 100 μm; 400×: 50 μm). (**K**–**N**) The levels of *Col I*, *Col III*, *α-SMA* and *TGF-β* mRNA in the heart tissue of mice were measured ($\bar{x}$ ± s, *n* = 3). (**O**,**P**) The mRNA levels of *GATA4* and *HIF-1α* in mouse heart tissue ($\bar{x}$ ± s, *n* = 3). (**Q**–**S**) The content of *Col I, Col III* and *α-SMA* protein in serum of mice with central muscle fibrosis ($\bar{x}$ ± s, *n* = 6). (**T**–**W**) Serum oxidative stress-related indicators HIF-1α, HO-1, ROS, SOD protein content in mice ($\bar{x}$ ± s, *n* = 6), * *p* < 0.05, ** *p* < 0.01, *** *p* < 0.001, *ns p* > 0.05.

To investigate the effects of various Hyp doses on cardiac function in MF mice, echocardiography and cardiac index evaluation were performed in mice. Echocardiography results (Figure 3A–E) revealed that, relative to the Control group, the Model group exhibited a significant reduction in both ejection fraction (EF) and shortening fraction (FS), along with a pronounced increase in left ventricular end-systolic diameter (LVIDs) and left ventricular end-diastolic diameter (LVIDd). Compared with the Model group, the LVIDs and LVIDd were significantly reduced in both the Hyp-L group and the Hyp-M group; the Hyp-H group exhibited a significant increase in EF and FS, while LVIDs and LVIDd diminished notably, and the improvement of each index was most obvious in all Hyp intervention groups. The Captopril group also exhibited a significant increase in FS and EF, as well as a significant decrease in LVIDs. In addition, the results shown in Figure 3F indicate that the cardiac index of the Model group was pronouncedly higher than that of the normal group. Compared with the Model group, the cardiac indices of the Captopril group, the Hyp-M group, and the Hyp-H group substantially declined. The cardiac index of the Hyp-H group decreased most notably, while the cardiac index of the Hyp-L group did not change significantly. Based on the results of this part, Hyp can effectively alleviate the cardiac function damage of MF mice by multi-dimensionally regulating cardiac structure and function indicators, among which the Hyp-H group has a particularly prominent effect on comprehensively reversing cardiac function damage.

### 2.3. Hyp Reduced Myocardial Injury and MF in MF Mice

To assess the cardioprotective effects of varying Hyp doses in MF mice, myocardial pathology was examined by H&E and Sirius red staining.

The results of H&E staining (Figure 3G,I) were used to evaluate histopathological changes in myocardial tissue across different groups. In the Control group, myocardial fibers were well organized and aligned, with clear striations and no obvious inflammatory cell infiltration. In contrast, the Model group showed disorganized myocardial fibers, disrupted architecture, and increased inflammatory cell infiltration (as indicated by the black arrow in Figure 3G), along with widened interstitial spaces, indicating the presence of myocardial injury. These changes were consistent with the increased H&E score compared with the Control group. The Hyp-H, Hyp-M and Captopril groups all showed varying degrees of histological improvement compared to the Model group: the arrangement of myocardial fibers was relatively more orderly, and the degree of inflammatory cell infiltration was reduced. In the Hyp administration group, the Hyp-H group demonstrated the most significant protective effect, with minimal inflammatory infiltration and a reduction in the number of inflammatory cells. This result was in line with the trend of the H&E score.

Sirius red staining (Figure 3H,J) was used to evaluate collagen deposition and myocardial fibrosis across different groups. In the Control group, myocardial fibers were arranged in an orderly manner, with sparse collagen fibers predominantly located in the interstitial space and minimal collagen deposition observed. In contrast, the Model group showed increased collagen deposition (as indicated by the black arrow in Figure 3H), with collagen fibers more prominently distributed in the interstitial spaces and surrounding myocardial bundles, indicating increased fibrosis. These changes were consistent with an increased collagen volume fraction compared with the Control group. Compared with the Model group, each administration group showed varying degrees of reduction in collagen deposition. Among these groups, the Hyp-H group demonstrated the most significant anti-fibrotic effect, characterized by reduced collagen deposition and relatively regular arrangement of myocardial fibers. Quantitative analysis further confirmed that the group showed the greatest reduction in collagen volume fraction.

In summary, Hyp intervention, especially high doses, proved highly effective in alleviating myocardial injury and attenuating fibrosis.

### 2.4. Hyp Inhibits the Expression of MF-Related Genes in MF Mice

This research further explored the effect of Hyp on the key factors of fibrosis in the heart tissue of MF mice from the perspective of gene expression regulation. The results of RT-qPCR are presented in Figure 3K–N. Relative to the Control group, the mRNA levels of *TGF-β*, *α-SMA*, *Col I* and *Col III* in the Model group were substantially upregulated. As opposed to the Model group, the gene expression of each group was downregulated to varying degrees: the expression of *α-SMA* mRNA in the Hyp low-dose group was significantly reduced; *Col I*, *Col III* and *α-SMA* mRNA in the Hyp-L group were pronouncedly decreased; and the mRNA levels of each index in the Hyp-H group and the Captopril group were markedly diminished. The results revealed that the Hyp-H group had the most significant improvement in the expression of MF-related genes. In summary, Hyp inhibits the transcription of fibrosis-related genes through multiple targets, which may play an anti-fibrotic role by blocking the fibrosis cascade of α-SMA transdifferentiation caused by activation of TGF-β signaling and excessive deposition of collagen (Col I/Col III). High-dose intervention has the best effect at the level of gene expression regulation.

### 2.5. Hyp Can Regulate the Expression Levels of GATA4 and HIF-1α Genes in the Cardiac Tissue of MF Mice

In order to further reveal the molecular regulation mechanism of Hyp reducing MF, especially the target at the signaling pathway level, the present study focused on the GATA4/HIF-1α pathway on the basis of clarifying the interaction between Hyp and GATA4, HIF-1α, and the inhibition of fibrosis-related gene expression by Hyp. Therefore, qRT-PCR was conducted to quantify *GATA4* and *HIF-1α* mRNA expression in the cardiac tissue of MF mice. As shown in Figure 3O,P, the Model group exhibited a marked decrease in *GATA4* mRNA expression but a pronounced upregulation in *HIF-1α* mRNA relative to the Control group. Relative to the Model group, all intervention groups showed markedly elevated *GATA4* mRNA expression; in contrast, *HIF-1α* mRNA levels significantly declined. Among them, the effect of the Hyp-H group on the expression of the two genes was the most obvious. The above results suggest that GATA4 and HIF-1α may be important molecular targets of Hyp against MF.

### 2.6. Hyp Regulates Serum Central Muscle Fibrosis and Oxidative Stress Levels in MF Mice

In order to further verify the protective effect of Hyp on MF mice from the peripheral level, this research used ELISA to detect the effect of Hyp intervention on the content of serum central muscle fibrosis-related proteins and oxidative stress-related molecules in MF mice. As depicted in Figure 3Q–S, the Model group exhibited significantly elevated serum levels of Col I, Col III and α-SMA protein relative to the Control group. Except for Col I in the Hyp-L group, the protein content of the above indexes in the other intervention groups was significantly lower than that in the Model group, and the decrease in each index in the Hyp-H group was the most significant. Relative to the Control group, the Model group exhibited pronounced increases in serum HIF-1α and ROS levels, alongside a significant reduction in HO-1 and SOD (Figure 3T–W). In comparison with the Model group, the serum HIF-1α and ROS protein levels in each intervention group notably declined; except for the Hyp-L group, the content of HO-1 in each administration group was considerably enhanced. Furthermore, the serum SOD protein levels in each treatment group were significantly lower than those in the model group. It can be seen that the Hyp-H group had the most obvious regulation of the above indicators. This result further revealed the regulatory effect of Hyp on systemic fibrosis and oxidative stress imbalance in MF mice from the peripheral level, and clarified the advantages of high-dose Hyp in regulating systemic fibrosis and oxidative stress. Based on these findings, a high dose of Hyp appears to be the optimal intervention for achieving cardioprotective effects against MF.

### 2.7. GATA4 Affects the Improvement of Hyp on Cardiac Function and Myocardial Cell Injury in MF Mice

In order to further explore whether GATA4 is a key regulator of Hyp’s protective effect, this research clarified the anti-fibrosis and cardiac function protective effects of Hyp (high-dose Hyp with the best effect confirmed above). Through AAV-GATA4 overexpressing GATA4, combined with echocardiography and serum myocardial injury marker spectrum enzyme detection, the effect of GATA4 on Hyp improving cardiac function and reducing myocardial cell injury was systematically evaluated.

**Figure 4:**
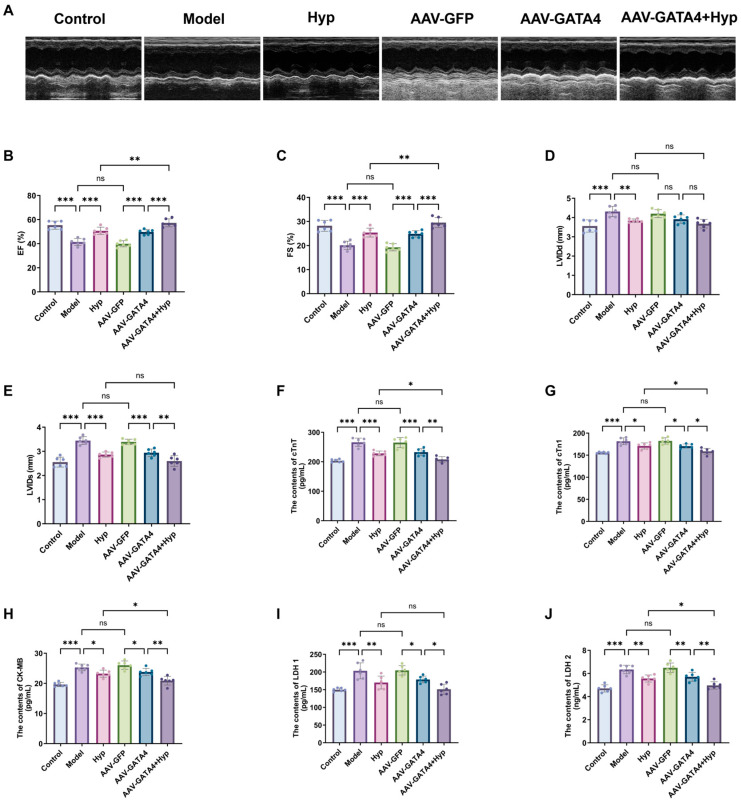
GATA4 affects the improvement of Hyp on cardiac injury in MF mice. (**A**) Representative diagram of echocardiography in mice. (**B**–**E**) EF, FS, LVIDd and LVIDs were detected by echocardiography in mice ($\bar{x}$ ± s, *n* = 6). (**F**–**J**) The contents of cTnT, cTn1, CK-MB, LDH 1 and LDH 2 in the serum of mice were measured ($\bar{x}$ ± s, *n* = 6). * *p* < 0.05, ** *p* < 0.01, *** *p* < 0.001, *ns p* > 0.05.

Echocardiography (Figure 4A–E) revealed markedly lower FS and EF in the Model group relative to the Control, alongside substantially elevated LVIDd and LVIDs. The Hyp group exhibited marked increases in EF and FS and significant decreases in LVIDs and LVIDd relative to the Model group. In the GATA4 overexpression intervention, as opposed to the AAV-GFP group, the EF and FS of the AAV-GATA4 group were substantially elevated, and the LVIDs declined. Compared with the AAV-GATA4 group, EF and FS in the AAV-GATA4+Hyp group were further markedly upregulated, and LVIDs notably decreased. The AAV-GATA4+Hyp group exhibited increased EF and FS relative to Hyp alone, while LVIDs were notably reduced. Figure 4F–J indicated an increase in serum LDH1, LDH2, CK-MB, cTnT, and cTnI in the Model group versus the Control group, and a decrease in these indices in the Hyp group, in contrast to the Model group. Compared with the AAV-GFP group, the levels of LDH1, LDH2, CK-MB, cTnT and cTnI in the AAV-GATA4 group were pronouncedly decreased. Relative to the AAV-GATA4 group, the indices of the AAV-GATA4+Hyp group were further reduced. The levels of LDH2, CK-MB, cTnT and cTnI in the AAV-GATA4+Hyp group were substantially lower than those in the Hyp group.

In summary, the results demonstrate that overexpression of GATA4 can independently improve cardiac function and reduce myocardial injury, suggesting that GATA4 is a key molecule for MF heart protection. The synergistic effect of GATA4 overexpression and Hyp combined intervention confirmed that the anti-MF protective effect of Hyp was partly dependent on its regulation and activation of GATA4, which clarified that GATA4 was the core target of Hyp to play a protective role.

### 2.8. GATA4 Affects the Regulation of Hyp on the Expression of Myocardial Markers in MF Mice

**Figure 5:**
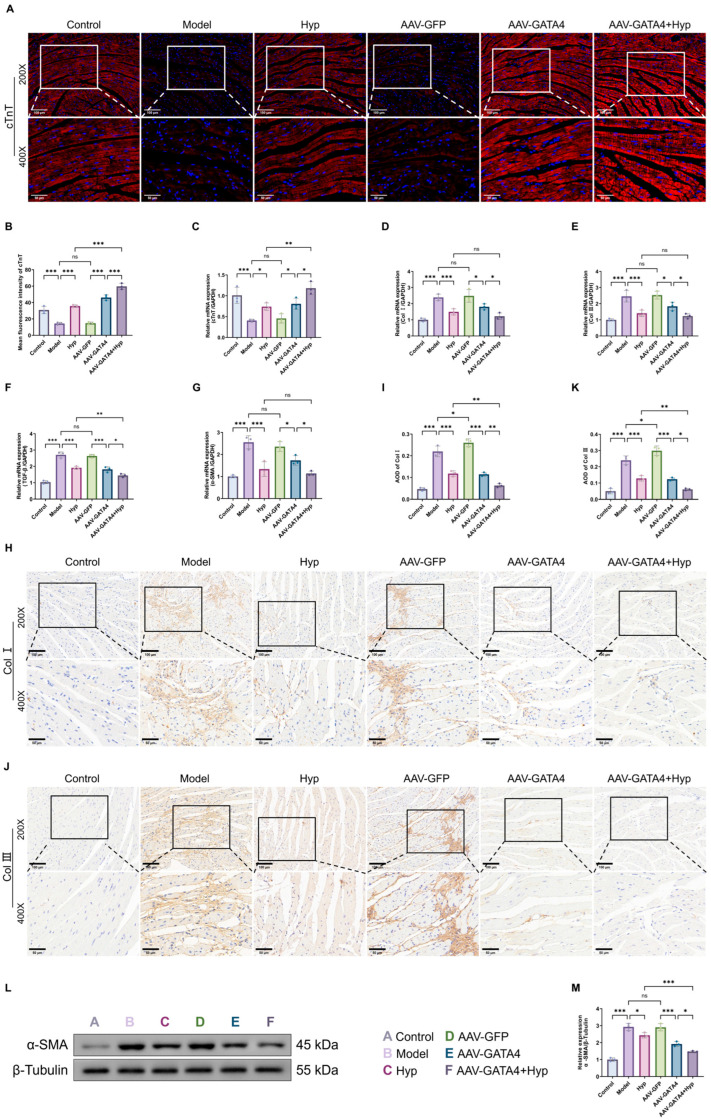
GATA4 affects the improvement of Hyp on cardiac injury in MF mice. (**A**,**B**) cTnT immunofluorescence representative map and cTnT positive cell count in mouse heart tissue ($\bar{x}$ ± s, *n* = 3), (200×: 100 μm; 400×: 50 μm). (**C**–**G**) The mRNA levels of *cTnT*, *Col I*, *Col III*, *TGF-β*, and *α-SMA* in mouse heart tissue ($\bar{x}$ ± s, *n* = 6). (**H**,**I**) The representative figure and average optical density of Col I immunohistochemical staining in mouse heart tissue ($\bar{x}$ ± s, *n* = 3). (**J**,**K**) The representative figure and average optical density of Col III immunohistochemical staining in mouse heart tissue. (**L**,**M**) The WB representation of α-SMA in mouse heart tissue and the relative expression of α-SMA protein ($\bar{x}$ ± s, *n* = 3). * *p* < 0.05, ** *p* < 0.01, *** *p* < 0.001, *ns p* > 0.05.

Subsequently, in order to further explore the effect of GATA4 on the expression of key molecules regulated by Hyp from myocardial markers, the current research focused on the core marker of myocardial cell injury and repair—cardiac troponin T (cTnT). Immunofluorescence staining was used to locate the cell distribution of cTnT protein, and qRT-PCR analysis was employed to systematically evaluate how GATA4 regulates Hyp-induced cTnT expression. Compared with the Control group, immunofluorescence staining of cTnT (Figure 5A,B) revealed significant structural and molecular alterations in the Model group. In the Control group, cTnT fluorescence was strong and uniformly distributed along well-organized myocardial fibers, showing clear continuity and intact cardiomyocyte morphology. In contrast, the Model group exhibited markedly reduced fluorescence intensity, accompanied by a disrupted and uneven distribution pattern. Myocardial fibers appeared disorganized and discontinuous, with fragmented fluorescence signals and loss of structural integrity, indicating severe cardiomyocyte injury. Meanwhile, RT-qPCR results (Figure 5C) demonstrated a significant downregulation of *cTnT* mRNA expression. Following Hyp intervention, the fluorescence intensity of cTnT was notably enhanced compared with the Model group. The signal distribution became more continuous and orderly, and myocardial fiber alignment was partially restored, suggesting improved cardiomyocyte structural integrity. This recovery was further supported by the upregulation of *cTnT* mRNA expression. In the AAV-GFP group and the Model group, there were no significant differences in the immunofluorescence intensity of cTnT and its mRNA level. Compared with the AAV-GFP group, the fluorescence in the AAV-GATA4 group was significantly enhanced, the fiber arrangement was clearer, and the structural continuity was also improved. Notably, the AAV-GATA4+Hyp group demonstrated the strongest fluorescence intensity and the most well-preserved myocardial architecture, characterized by densely arranged, continuous, and uniformly distributed cTnT signals. Furthermore, the fluorescence intensity in the AAV-GATA4+Hyp group was significantly higher than that in the Hyp group alone, indicating a synergistic effect of GATA4 overexpression and Hyp treatment in preserving cardiomyocyte integrity and promoting myocardial repair.

### 2.9. GATA4 Affects the Expression Level of Central Muscle Fibrosis-Related Indicators in Cardiac Tissue of MF Mice Regulated by Hyp

This investigation further explored the effect of GATA4 on the anti-fibrotic effect of Hyp from the multi-level of gene transcription–protein expression–tissue localization. As shown in Figure 5D–G, myocardial expression of *Col I*, *Col III*, *TGF-β* and *α-SMA* mRNA was elevated in the Model group relative to the Control group. Expression of these genes was substantially lower in the Hyp group than in the Model group; similarly, mRNA levels of *Col I*, *Col III*, *TGF-β* and *α-SMA* were pronouncedly reduced in the AAV-GATA4 group compared to the AAV-GFP group. The combined intervention of AAV-GATA4+Hyp further reduced the contents of the four indicators. Western blot (Figure 5L,M) revealed that α-SMA protein expression was significantly elevated in the Model group versus the Control group, and that of the Hyp group was substantially lower than in the Model group. The α-SMA protein expression was significantly diminished in the AAV-GATA4 cohort compared to the AAV-GFP group. The AAV-GATA4+Hyp group was further lower than the AAV-GATA4 group, and the decrease was more significant than the Hyp group alone. Figure 5H–K show the immunohistochemical staining of Col I and Col III. In the Control group, Col I and Col III staining were weak and sparsely distributed, mainly confined to the interstitial and perivascular regions, indicating normal ECM turnover and structural homeostasis. In contrast, the Model group exhibited markedly increased positive-staining intensity for both Col I and Col III. Dense brown staining was widely distributed throughout the myocardial interstitium, particularly between myocardial fiber bundles and around blood vessels. The collagen fibers become thickened, aggregated and arranged irregularly, while the inter-fiber spaces expand, indicating a severe remodeling of the ECM and MF. Quantitative analysis (Figure 5I,K) further confirmed a significant increase in the collagen fibers becoming thickened, aggregated and irregularly arranged, accompanied by an expansion of the inter-fiber spaces, indicating severe extracellular matrix remodeling and MF of Col I and Col III in the Model group compared with the Control group. Following Hyp intervention, the expressions of Col I and Col III were markedly reduced. The density and continuity of collagen staining were significantly attenuated, with a more scattered and less organized distribution pattern. The interstitial spaces appeared less expanded, and perivascular collagen accumulation was alleviated, suggesting partial reversal of fibrotic remodeling. Compared with the AAV-GFP group, the AAV-GATA4 group showed a substantial reduction in Col I and Col III expression, with decreased staining intensity and improved structural organization of myocardial fibers. Notably, the AAV-GATA4+Hyp group exhibited the lowest levels of collagen deposition, characterized by faint and discontinuous staining, minimal interstitial expansion, and near restoration of normal myocardial architecture. Furthermore, compared with the Hyp group alone, the combined AAV-GATA4+Hyp intervention resulted in a more pronounced reduction in collagen accumulation, indicating a synergistic anti-fibrotic effect. Consistently, quantitative analysis demonstrated that the AOD values of Col I and Col III were significantly decreased in the combined treatment group. These findings suggest that GATA4 plays a critical regulatory role in Hyp-mediated attenuation of MF, contributing to improved myocardial structural remodeling.

### 2.10. Hyp Regulates GATA4/HIF-1α Pathway Expression in MF Mice

**Figure 6:**
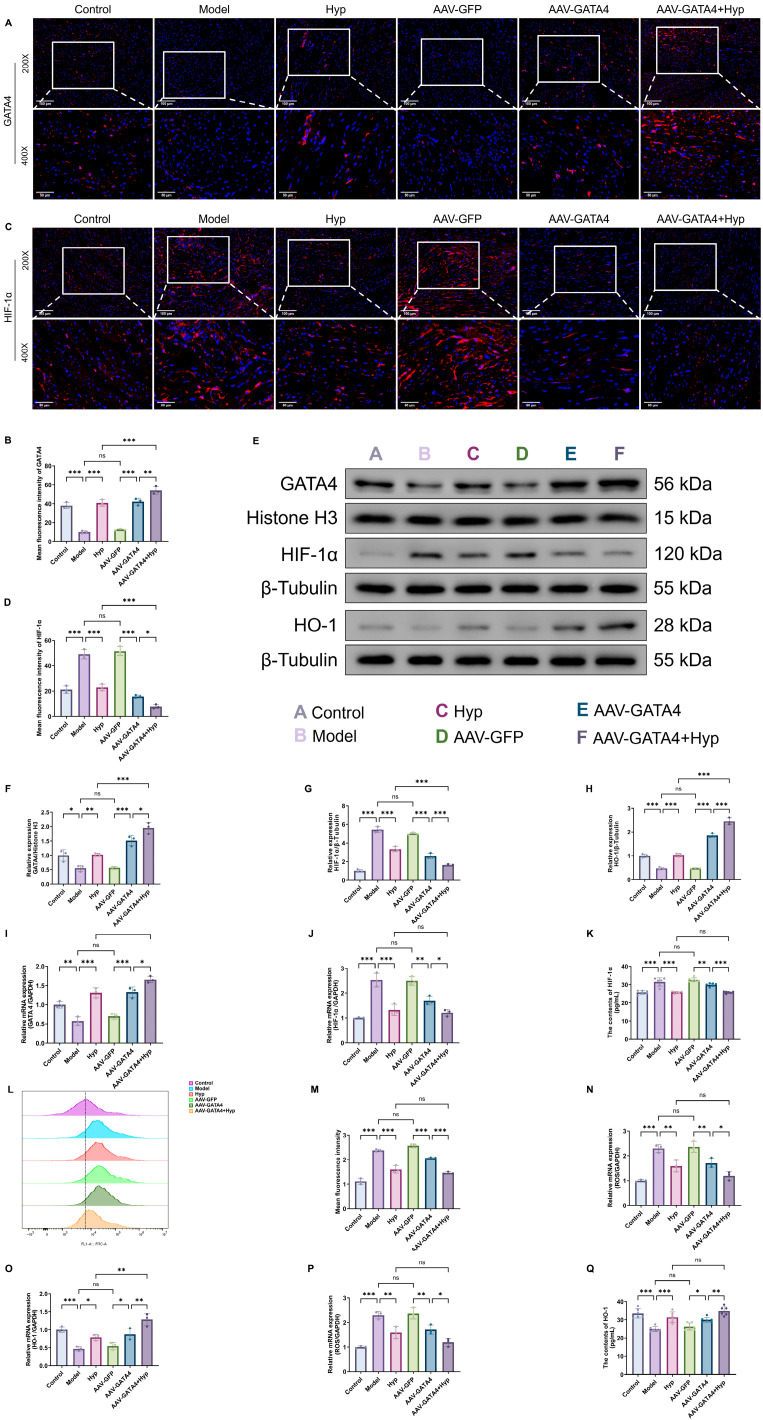
Hyp improves MF in mice through GATA4/HIF-1α pathway. (**A**,**B**) GATA4 immunofluorescence staining representative map and fluorescence quantification of mouse heart tissue ($\bar{x}$ ± s, *n* = 3) (200×: 100 μm; 400×: 50 μm). (**C**,**D**) The HIF-1α immunofluorescence staining representative map and fluorescence quantification of mouse heart tissue ($\bar{x}$ ± s, *n* = 3) (200×: 100 μm; 400×: 50 μm). (**E**) The representative figures of GATA4, HIF-1α and HO-1 WB in mouse heart tissue. (**F**–**H**) The relative expression levels of GATA4, HIF-1α and HO-1 proteins ($\bar{x}$ ± s, *n* = 3). (**I**,**J**) The mRNA levels of *GATA4* and *HIF-1α* in mouse heart tissue ($\bar{x}$ ± s, *n* = 3). (**K**) The content of HIF-1α protein in serum of mice ($\bar{x}$ ± s, *n* = 3). (**L**,**M**) ROS flow cytometry representative figure and protein content in mouse cardiomyocytes ($\bar{x}$ ± s, *n* = 3). (**N**,**O**) The levels of *ROS* and *HO-1* mRNA in mouse heart tissue ($\bar{x}$ ± s, *n* = 3). (**P**,**Q**) The content of ROS and HO-1 protein in serum of mice ($\bar{x}$ ± s, *n* = 3). * *p* < 0.05, ** *p* < 0.01, *** *p* < 0.001, *ns p* > 0.05.

This study investigated the impact of Hyp on GATA4/HIF-1α pathway expression in an MF mouse model. This study systematically analyzed the protein and mRNA levels of *GATA4* and *HIF-1α* in both cardiac tissue and serum. The results of WB and RT-qPCR (Figure 6E–G,I,J) revealed that compared to the Control group, the expression levels of GATA4 protein and mRNA in the heart tissue of the Model group were significantly diminished, while the expression levels of HIF-1α protein and mRNA were pronouncedly increased. After Hyp intervention, GATA4 levels increased substantially, whereas HIF-1α levels decreased markedly. Overexpressing GATA4 in cardiac tissue significantly increased GATA4 expression while decreasing HIF-1α expression, compared to the AAV-GFP group. The combined intervention of AAV-GATA4+Hyp further augmented the level of GATA4 and decreased the expression of HIF-1α. Compared to the Hyp group alone, the expression of GATA4 protein in the heart of the AAV-GATA4+Hyp combined intervention group was higher, and the levels of HIF-1α protein and mRNA were lower. The data in Figure 6K confirmed elevated serum HIF-1α protein levels in the Model group relative to the Control group. In comparison to the Model group, the blood HIF-1α levels in the Hyp group were dramatically reduced. Serum HIF-1α protein level was markedly reduced in the AAV-GATA4 group relative to the AAV-GFP group. The AAV-GATA4+Hyp group was further downregulated compared with the AAV-GATA4 group. Immunofluorescence staining of GATA4 and HIF-1α in myocardial tissue (Figure 6A–D) revealed that in the Control group, GATA4 fluorescence was moderately expressed and uniformly distributed within cardiomyocytes, predominantly localized in the nuclei, with clear alignment along well-organized myocardial fibers. In contrast, HIF-1α exhibited low basal expression, with weak and scattered fluorescence signals. In the Model group, a marked reduction in GATA4 fluorescence intensity was observed, accompanied by a disrupted and discontinuous distribution pattern. GATA4-positive signals were sparse and irregular, indicating impaired transcriptional activity and structural disorganization of cardiomyocytes. Conversely, HIF-1α fluorescence was significantly enhanced, with strong and widespread signals distributed throughout the myocardium, particularly along disorganized fiber regions. Following Hyp intervention, GATA4 fluorescence intensity was notably increased, with a more continuous and uniform nuclear distribution pattern, and partial restoration of myocardial fiber alignment. Meanwhile, HIF-1α fluorescence intensity was markedly reduced, with decreased signal density and a more restricted distribution. The AAV-GFP group showed no significant improvement compared with the Model group. In contrast, the AAV-GATA4 group exhibited a pronounced increase in GATA4 fluorescence, characterized by stronger and more continuous nuclear localization signals, along with a concomitant reduction in HIF-1α expression. Notably, in the AAV-GATA4+Hyp combined intervention group, GATA4 fluorescence reached the highest level, displaying dense, uniformly distributed nuclear signals aligned with well-organized myocardial fibers. Simultaneously, HIF-1α expression was further suppressed, with only weak and scattered fluorescence observed. Furthermore, compared with the Hyp group alone, the combined AAV-GATA4+Hyp treatment resulted in a more pronounced upregulation of GATA4 and a greater reduction in HIF-1α expression, indicating a synergistic regulatory effect. Based on the above results, it can be seen that Hyp can effectively upregulate the expression of GATA4 in the heart of MF mice and inhibit the expression of HIF-1α, while exogenous overexpression of GATA4 can further enhance the regulation of Hyp on this pathway, indicating that GATA4/HIF-1α signaling pathway is one of the key molecular mechanisms for Hyp to reduce MF, and GATA4 plays an important positive regulatory role in it.

### 2.11. Hyp Regulates the Level of Oxidation in MF Mice Through GATA4/HIF-1α Pathway

**Figure 7:**
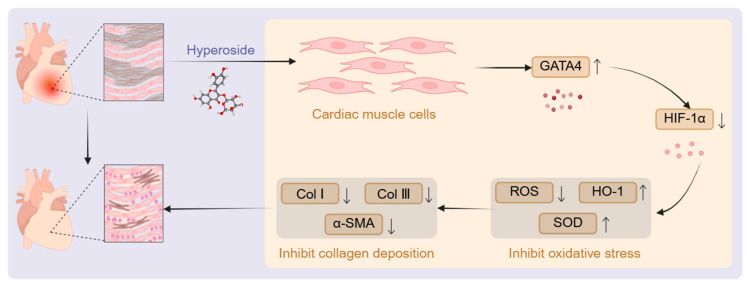
Hyp improves oxidative stress in MF through the GATA4/HIF-1α pathway. MF can cause pathological damage to the heart muscle and subsequently affect cardiac function. Hyp can exert a protective effect on the heart by targeting cardiac muscle cells and regulating the GATA4/HIF-1α pathway to inhibit oxidative stress and reduce collagen deposition. Eventually, this can alleviate pathological damage to the heart and improve MF.

In order to further elucidate the specific mechanism of Hyp regulating the oxidation level in MF mice through the GATA4/HIF-1α pathway, the levels of ROS and HO-1 in the heart and serum were measured in the study. Flow cytometry (Figure 6L,M) indicated that ROS levels in myocardial cells were significantly elevated in the Model group relative to controls, but were notably attenuated following Hyp intervention. Relative to the AAV-GFP group, the content of myocardial ROS protein in the AAV-GATA4 group was also substantially reduced, while the AAV-GATA4+Hyp group was further reduced compared with the AAV-GATA4 group. The results of WB (Figure 6E,H) showed that the expression of HO-1 protein in the heart of the Model group was significantly lower than that of the Control group, and the expression was pronouncedly elevated after Hyp intervention. The AAV-GATA4 group exhibited an elevated HO-1 protein level compared to the AAV-GFP group. The AAV-GATA4+Hyp group was further increased than the AAV-GATA4 group, and was pronouncedly higher than the Hyp group alone. As illustrated in Figure 6N,O, the RT-qPCR results revealed that the Model group exhibited a significant upregulation of *ROS* mRNA levels in cardiac tissue, accompanied by a notable downregulation of *HO-1* mRNA expression. Hyp intervention can reduce *ROS* mRNA and increase *HO-1* mRNA. Compared with the AAV-GFP group, *ROS* mRNA diminished and *HO-1* mRNA elevated in the AAV-GATA4 group. Compared with the AAV-GATA4 group, *ROS* mRNA was further decreased in the AAV-GATA4+Hyp group, but *HO-1* mRNA pronouncedly declined. Relative to the Hyp group, the expression of *HO-1* mRNA in the hearts of the AAV-GATA4+Hyp group was notably elevated. It can be seen from Figure 6P,Q that the serum ROS protein level in the Model group was notably augmented, and the HO-1 protein level was substantially diminished. Hyp reduced serum ROS and upregulated HO-1. The AAV-GATA4 group demonstrated a reduction in serum ROS and an increase in HO-1 levels compared to the AAV-GFP group. Relative to the AAV-GATA4 group, the serum ROS in the AAV-GATA4+Hyp group was further reduced, but the HO-1 protein level exhibited a significant downward trend. The above results indicate that Hyp is involved in the regulation of the GATA4/HIF-1α pathway to exert antioxidant effects, thereby reducing myocardial oxidative damage (Figure 7).

## 3. Discussion

As a complex pathological process driven by multiple factors, the treatment of MF is still facing a significant dilemma: once the disease is formed, it is difficult to reverse, and the existing methods are mostly focused on delaying progress rather than achieving repair [20,21,22]. There is a close regulatory relationship between its occurrence and development and oxidative stress, which together constitute a vicious circle to promote cardiac remodeling. Oxidative stress promotes fibrosis through multiple molecular pathways. The overproduction of ROS can directly stimulate cardiac fibroblasts, facilitating their conversion into highly secretory myofibroblasts and enhancing the synthesis and deposition of ECM proteins [23]. Studies have demonstrated that ROS can enhance the transduction efficiency of TGF-β signals by oxidative modification of the TGF-β receptor or inhibition of its negative regulators, thereby upregulating the expression of fibrosis markers such as α-SMA [24,25]. Recent studies have also found that oxidative stress can affect the process of fibrosis through epigenetic regulation. For example, ROS-induced histone modifications and DNA methylation changes can permanently activate the expression program of profibrotic genes [26]. At the same time, oxidative stress-induced mitochondrial dysfunction and endothelial–mesenchymal transition (EndMT) are also involved in the development of MF [27]. Therefore, intervention against oxidative stress is not only a strategy to reduce MF, but also a key link in blocking the process of fibrosis.

Hyp, a natural flavonoid glycoside, has been documented to exhibit many pharmacological properties, including antioxidative, anti-inflammatory, antifibrotic, and cardiovascular protective effects. In terms of anti-oxidation, Hyp can effectively remove excess ROS and RNS in the body, and stimulate essential natural antioxidant enzymes, including SOD and GSH-Px, thereby reducing oxidative stress damage. This feature has laid an important foundation for its cardiovascular protection [28,29]. Existing studies have confirmed that Hyp can reduce MI-induced oxidative stress in cardiomyocytes by activating the JAK2/STAT3 pathway [5]. In addition, Hyp also targets the NOX/ROS/NLRP3 signaling pathway to prevent doxorubicin-induced cardiotoxicity [12]. In addition, Hyp can impede the progression of pulmonary fibrosis by reducing oxidative stress levels in animals with bleomycin-induced lung fibrosis [30]. Hyp can also reduce liver fibrosis induced by carbon tetrachloride [31]. These findings suggest that Hyp can reverse fibrosis by inhibiting oxidative stress.

However, the current research focuses on the improvement of Hyp on myocardial infarction, drug-induced heart injury and myocardial hypertrophy caused by Hyp, but the therapeutic effect on MF is still unclear. To this end, this investigation systematically explored the effect of Hyp on an ISO-induced MF mouse model, and multi-dimensional experiments verified the anti-fibrotic effect of Hyp. As evidenced by the results, Hyp intervention effectively restored cardiac systolic and diastolic function, with a concomitant improvement in cardiac index. More importantly, it can significantly reduce myocardial injury and myocardial interstitial collagen deposition and simultaneously downregulate the expression of key fibrosis markers, α-SMA, Col I and Col III, at the gene and protein levels. This result is consistent with previous studies [5,11,23]. This multi-level improvement effect suggests that Hyp may act on the upstream regulatory hub of the fibrosis process, rather than merely antagonizing the downstream terminal effect. It is worth noting that Hyp significantly reduced ROS and HIF-1α to reduce oxidative stress levels in tissues and systemic circulation while exerting anti-fibrosis effects, and increased the antioxidant enzyme HO-1 to enhance endogenous antioxidant defense capabilities. This suggests that Hyp may block the vicious cycle of ‘oxidative stress-fibrosis’ by balancing the state of oxidative stress. At the same time, we detected that Hyp can regulate the gene levels of *GATA4* and *HIF-1α* in the visceral tissues of MF mice, suggesting that GATA4 and HIF-1α may be important molecular targets for Hyp anti-MF. In terms of dose effect, this research identified that high dose Hyp (36 mg·kg^−1^) as the optimal dosage for enhancing cardiac function, reducing fibrosis and regulating gene expression, which provided a dose reference for clinical application.

GATA4 and HIF-1α are two key transcriptional regulators in cardiac development and pathological remodeling. There is a close interaction between the two, which constitutes an important regulatory axis-GATA4/HIF-1α pathway, which plays a central role in maintaining cardiac homeostasis and participating in the pathogenesis of myocardial diseases. In the process of MF, when the expression of GATA4 decreases or is dysfunctional, its inhibitory effect on HIF-1α is weakened, resulting in abnormal accumulation of HIF-1α. This, in turn, upregulates the expression of prooxidase, enhancing ROS production and, not only directly activating cardiac fibroblasts and promoting ECM deposition, but also further activating the classical profibrotic pathway to form an ‘oxidative stress-fibrosis’ positive feedback loop. In conclusion, the GATA4/HIF-1α pathway links transcriptional regulation with oxidative stress, hypoxia response and fibrosis process, providing an integrated perspective for understanding the molecular mechanism of MF. Intervention against this pathway may become a new strategy to reverse or delay MF. Therefore, this paper explores whether the GATA4/HIF-1α pathway is one of the possible mechanisms of Hyp in the treatment of MF. Firstly, this study revealed the stable binding of Hyp to the key targets of the GATA4/HIF-1α pathway through molecular docking and kinetic simulation. The binding energy was lower than −5.0 kcal·mol^−1^, indicating high affinity. In the 100 ns all-atom MD simulation process, Hyp maintained stable interaction with GATA4 and HIF-1α targets; low RMSD fluctuation, stable SASA value, concentrated free energy, and high conformational stability supported the reliability of the binding mode. This result suggests that Hyp may directly target the GATA4/HIF-1α pathway. Furthermore, this paper verified this hypothesis by a GATA4 overexpression experiment. First of all, GATA4 is a central transcription factor essential for cardiac development and functional maintenance. Overexpression of GATA4 can independently improve cardiac function and reduce myocardial enzyme levels to reduce myocardial cell injury, promote the expression of myocardial marker cTnT to repair damage and reduce the expression of α-SMA, Col I and Col III to improve MF. This is consistent with previous studies: GATA4 deficiency aggravates cardiac hypertrophy and fibrosis, while overexpression of GATA4 can reduce cardiac remodeling by inhibiting fibroblast activation [16]. It is worth noting that GATA4 overexpression, combined with Hyp intervention, demonstrated a synergistic effect. Secondly, the molecular mechanism research found that Hyp activated the GATA4/HIF-1α pathway by upregulating GATA4 and downregulating HIF-1α gene and protein expression. Further flow cytometry, WB, RT-qPCR and ELISA collectively demonstrated that Hyp was involved in the regulation of the GATA4/HIF-1α pathway to regulate oxidative stress, thereby reducing myocardial oxidative damage. This is different from its traditional cognition as a simple free radical scavenger, indicating that Hyp may break the oxidative stress loop that promotes fibrosis by regulating the overall redox signal network of cells. This antioxidant effect may be related to the chemical structure of Hyp, whose flavonoid group can directly scavenge free radicals, but this investigation emphasizes the integrity of pathway regulation. In summary, GATA4/HIF-1α is a key target for Hyp to exert the protective effect of MF, and the two achieve cardiac protection by synergistically regulating fibrosis, oxidative stress and cardiac function.

Intercellular communication among distinct myocardial cell populations plays a crucial role in cardiac injury and repair [32]. Although MF has traditionally been attributed to fibroblast activation, accumulating evidence suggests that an increasing amount of cardiomyocytes indicates regulated fibroblast behavior through autocrine and paracrine signaling, thereby contributing to this process [33,34,35]. Based on these findings, we hypothesize that cardiomyocyte-specific expression of GATA4 may regulate the fibroblast activation through paracrine mechanisms. In this study, cardiomyocyte-specific overexpression of GATA4 in the MF state reduced the release of the profibrotic factor TGF-β, which may, in turn, attenuate fibroblast activation by suppressing downstream SMAD signaling [36]. Furthermore, oxidative stress is a key driver of fibroblast activation. The research results indicate that cardiomyocyte-specific overexpression of GATA4 may reduce ROS levels and enhance HO-1-mediated antioxidant signaling, thereby attenuating oxidative stress in fibroblasts. Overall, these findings indicate that GATA4 in cardiomyocytes exerts an antifibrotic effect by coordinating paracrine regulation of profibrotic signaling and oxidative stress, thereby maintaining fibroblasts in a relatively quiescent state.

The synergistic effect of Hyp combined with GATA4 in the present study indicated that the anti-MF effect of Hyp was partially dependent on the upregulation and activation of GATA4, rather than a completely independent mechanism, which was consistent with the characteristics of GATA4 as a ‘hub’ to integrate multiple protective signals. The mechanism of Hyp inhibiting oxidative stress and fibrosis by regulating the GATA4/HIF-1α pathway is of great innovation. On the one hand, overexpression of GATA4 can enhance the inhibitory effect of Hyp on Col I/III, α-SMA, etc., suggesting that GATA4 may directly bind to the promoter region of fibrosis-related genes to inhibit their transcription, or indirectly regulate them through downstream effector molecules, thereby enhancing the anti-fibrosis effect [16]. At the mechanism level, Hyp effectively reversed the oxidative stress state in MF by upregulating the expression of GATA4 and downregulating the level of HIF-1α. GATA4 may regulate by inhibiting the transcription of HIF-1α or promoting its degradation, thereby reducing ROS production and activating the HO-1 antioxidant pathway. This provides a new perspective for understanding the pathophysiological process of MF from early stress response to late irreversible structural remodeling, and also suggests that targeting both oxidative stress and specific transcriptional regulatory nodes may be a more fundamental intervention strategy.

However, this study has several limitations. First, the present study was conducted exclusively in vivo, and no in vitro experiments were performed to further validate the underlying cellular mechanisms. Second, although AAV-mediated overexpression was used to investigate the role of GATA4, complementary approaches such as gene knockout models or pharmacological inhibitors were not employed to provide reverse validation. Therefore, future studies should focus on combining in vitro experiments with genetic or pharmacological approaches to further elucidate the precise mechanisms underlying the observed effects.

## 4. Materials and Methods

### 4.1. Molecular Docking

The Hyp structure was optimized by Chem3D and converted into pdb format. After obtaining the crystal structure of the target protein (GATA4, PDB ID: 8VG0; HIF-1α, PDB ID: 8HE0) from RCSB PDB, the ligand and other components in the original structure were removed by PyMol. The docking site was determined using the POCASA server, and the docking area was defined based on the top-ranked predicted binding pocket. The grid box was centered on the geometric center of the predicted pocket, with a box size of 24 × 24 × 24 Angstrom to fully cover the binding cavity and the surrounding key residues. AutoDock Vina 1.1.2 was used to complete hydrogen bond optimization and charge calculation. A genetic algorithm was used for global search, and a default scoring function was used for binding energy calculation. The docking results were visualized by PyMol 2.5.x for the hydrogen bond network and binding conformation.

### 4.2. Molecular Dynamics Simulation

In this study, GROMACS 2023.2 was used to perform a 100 ns all-atom molecular dynamics simulation on the complex. Based on the CHARMM36 force field parameterization system, the ligand topology was generated by CGenFF. In the pretreatment stage, the energy was minimized by the steepest descent method (maximum residual force < 1000 kJ/mol/nm), followed by 100 ps NVT (300 K, V-rescale thermostat) and NPT (1 bar, C-rescale potentiostat) equilibrium, during which position restrictions were imposed on the protein and ligand. Production runs at 300 K and 1 bar for 100 ns, with trajectories saved every 100 ps. The stability of the complex was evaluated by RMSD, the number of hydrogen bonds and SASA, and the free energy landscape map was constructed with Protein–Ligand RMSD and centroid distance as reaction coordinates to calculate the Gibbs free energy change [37].

### 4.3. Animal Experiment

#### 4.3.1. Animals

Male C57BL/6 mice (21–23 g and 6–8 weeks) with SPF status were procured from Shanghai Slake Experimental Animal Co., Ltd. (Shanghai, China, Production license number: SCXK (Shanghai) 2022-0004). All animal experiments were conducted in strict compliance with the ethical guidelines and regulatory frameworks established by the respective nations governing laboratory animal welfare, and in accordance with the requirements of ARRIVE 2.0 guidelines, and sanctioned by the Animal Research Ethics Board of Zhejiang Chinese Medical University (Approval number: IACUC-20240701-12). Before the experiment began, these mice were acclimated for 7 days in an environment with a temperature of 20–25 °C, an environmental humidity of 50–60%, and a 12/12 h light/dark cycle. During this period, the mice were allowed to eat and drink freely. Each cage contained 5 mice. At the end of the experiment, Zoletil 50 (Virbac AH, Inc., Fort Worth, TX, USA) (200 mg/kg) was injected into the mice’s abdominal cavities, and then they were euthanized by bloodletting. All efforts were made to minimize animal suffering and the number of animals used.

#### 4.3.2. Animal Model Construction and Group Administration

Isoproterenol (ISO, 51-30-9, Aladin, Shanghai, China) was dissolved in normal saline and injected subcutaneously at a dosage of 50 mg·kg^−1^, once a day for 5 consecutive days to establish a mouse MF model.

In the first experiment, mice were allocated into the following groups: Control group, Model group, Low dose Hyp group (Hyp-L, 9 mg·kg^−1^, 482-36-0, Lemeitian, Chengdu, China), Medium Hyp dose group (Hyp-M, 18 mg·kg^−1)^, High Hyp dose group (Hyp-H, 36 mg·kg^−1^), Positive drug group (Captopril, 25 mg·kg^−1^), with each group consisting of 12 animals. The Control and Model groups received an equal volume of normal saline via daily intragastric administration for 14 consecutive days.

**Figure 8:**
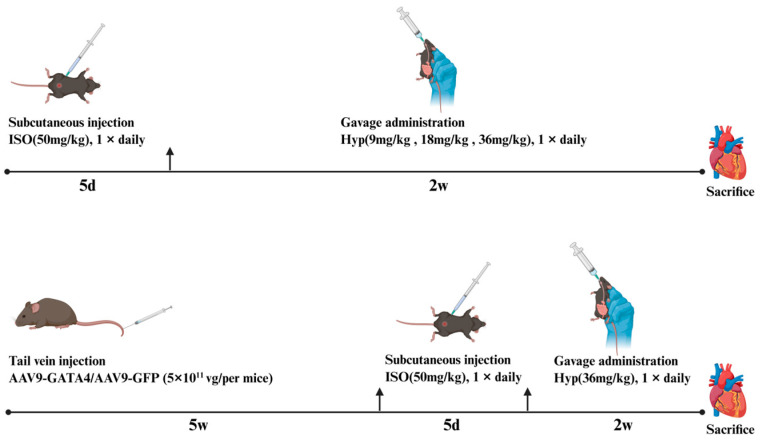
Flow chart of the experiment.

In the second experiment, mice were allocated into the following groups: Control group, Model group, Hyp group, AAV9-GFP group, AAV9-GATA4 group, AAV9-GATA4+Hyp group, with each group consisting of 12 animals. The AAV9-GATA4 group and AAV9-GATA4+Hyp group were injected with AAV9-GATA4 (5 × 10^11^ VG per animal, Vigenebio, Rockville, MA, USA) by tail vein, and the AAV9-GFP group was injected with AAV9-GFP (5 × 10^11^ VG per animal, Vigenebio, Rockville, MA, USA) by tail vein as a blank plasmid control group. After 5 weeks of AAV9 injection, the model group, Hyp group, AAV9-GFP group, AAV9-GATA4 group and AAV9-GATA4+Hyp group were subcutaneously injected with 50 mg/kg ISO for 5 consecutive days to construct the MF model. Subsequently, mice in the Hyp group and the AAV9-GATA4+Hyp group were given intragastric administration at a dose of 36 mg·kg^−1^ for 14 days. Figure 8 presents the experimental process.

#### 4.3.3. Echocardiogram

The mice in each group were measured by echocardiography on the 14th day of administration. Mice received isoflurane anesthesia, with cardiac imaging performed via small animal Doppler ultrasound. Then, the cardiac systolic and diastolic motion trajectories were recorded in M mode, and three consecutive cardiac cycles were recorded along the long axis. EF, FS, LVIDd and LVIDs were detected to analyze the cardiac function of the model mice.

#### 4.3.4. Heart Weight Index

After the cardiac function test, the mice’s hearts were weighed. After taking the heart, non-myocardial tissue was removed. The heart was washed to remove blood, then dried with filter paper. Afterward, the whole heart was weighed. Heart weight index (HW/BW, heart weight/body weight) = heart weight (mg)/body weight (g).

#### 4.3.5. Hematoxylin and Eeosin (H&E) Staining

Mouse heart tissues were fixed in 4% paraformaldehyde at room temperature for 24 h, followed by routine dehydration, paraffin embedding, and sectioning at 4 μm thickness. Sections were deparaffinized in xylene and rehydrated through a graded ethanol series. The sections were stained with hematoxylin for 10 min, differentiated in 1% acid alcohol, and blued in water. Subsequently, sections were counterstained with eosin for 1 min, followed by dehydration, clearing, and mounting with neutral resin. Histopathological changes were observed under a light microscope. Tissue injury was evaluated in a blinded manner using a semi-quantitative scoring system based on inflammatory cell infiltration and tissue damage. Multiple random fields were analyzed for each section. Scoring criteria were defined as follows: normal tissue with no detectable injury or inflammatory cell infiltration was scored as 0; slight tissue damage with occasional inflammatory cell infiltration was scored as 1; mild tissue damage with localized inflammatory cell infiltration was scored as 2; moderate tissue damage with extensive inflammatory cell infiltration was scored as 3; severe tissue damage with widespread inflammatory cell infiltration was scored as 4 [38].

#### 4.3.6. Sirius Red Staining

Paraffin-embedded heart sections were deparaffinized and rehydrated as described above, and sectioned at 4 μm thickness. Sections were stained with Sirius Red solution for 8 min, then rinsed with anhydrous ethanol and mounted. Under a light microscope, collagen fibers appeared red, while other tissue components were stained yellow. Quantitative analysis of collagen content was performed using ImageJ software (Version 1.54h) by calculating the percentage of collagen-positive area relative to the total tissue area.

#### 4.3.7. ELISA

Blood from all groups was gathered for 60 min, then centrifuged at 4500 r/min for 15 min under ambient conditions, after which the supernatant was extracted. The expression levels of cTnT, cTn1, CK-MB, LDH1, LDH2, ROS, SOD, HIF-1α, α-SMA, Col I, and Col III in the serum of mice were determined by ELISA kit instructions (Jiangsu Meibiao Biotechnology, Yancheng, China). All assays were performed based on a double-antibody one-step sandwich ELISA principle.

#### 4.3.8. Western Blot

The cardiac tissue was extracted, to which RIPA lysis buffer was added (1 mL of RIPA lysis buffer is added for every 100 mg of tissue) for homogenization, and then centrifuged to obtain the supernatant. The protein concentrations of the standard curve and each sample were determined using the BCA method. Then, the target protein was separated by electrophoresis and transferred to a PVDF membrane. The membrane was then blocked for 1 h in TBST containing 5% skim milk. Following overnight incubation at 4 °C, the membrane was probed with primary antibodies (1:1000) against: GATA4 (307823, Abcam, Cambridge, UK), HIF-1α (48058, CST, Danvers, MA, USA), HO-1 (86806T, CST, Danvers, MA, USA), a-SMA (AF1032, Affinity Biosciences, Cincinnati, OH, USA), Histone H3 (BF9211, Affinity Biosciences, Cincinnati, OH, USA) and β-Tubulin (T0023, Affinity Biosciences, Cincinnati, OH, USA). Following this, the membrane underwent treatment with secondary antibody (1:5000) at 25 °C for 2 h. To wrap things up, we determined the target protein’s relative expression level by dividing the gray value of its corresponding band by the gray value of either β-Tubulin or Histone H3, which served as our internal reference standards.

#### 4.3.9. RT-qPCR

The total RNA from the heart tissues of mice was extracted using the TRIzol method, and its concentration and purity were determined. Subsequently, the RNA was reverse-transcribed into cDNA using a reverse transcription kit (CW2020M, Kangwei Century, Taizhou, China). Using the reverse-transcribed cDNA as the template, a qPCR reaction was performed with primers, with GAPDH as the internal reference. Reverse transcription was conducted under the following conditions: 42 °C, 15 min and 85 °C, 5 min. Thoroughly mixed samples were loaded in a 96-well plate and amplified under the following conditions: 95 °C, 10 min, denaturation; 95 °C, 15 s; 60 °C, 60 s, 40 cycles. The primer sequences are listed in Table 2 (Sangon Biotech, Shanghai, China). The relative expression levels of each indicator mRNA were calculated using the 2^−ΔΔCt^ method [39]. The formula is as follows:

$$\Delta \Delta Ct={(Ct}_{Sample}-{Ct}_{GAPDH})-({Ct}_{Control}-{Ct}_{GAPDH})$$

#### 4.3.10. Flow Cytometry

Myocardial tissues were rinsed with ice-cold buffer, and the left ventricle was isolated and minced into small fragments as thoroughly as possible. The tissue was then transferred to a centrifuge tube containing 5 mL of digestion solution (1.6 mg/mL RPMI 1640 medium (C11875500BT, Gibco, Waltham, MA, USA) supplemented with 0.2 mg/mL collagenase I (1904MG100, BioFroxx, Einhausen, Germany)) and incubated at 37 °C with gentle agitation. After 1 h of digestion, the tissue suspension was filtered through a 70 μm nylon mesh and centrifuged at 1500 rpm for 5 min at 4 °C. The supernatant was discarded, and the cell pellet was resuspended in 200 µL of red blood cell lysis buffer (MA0165, MeilunBio, Dalian, China) and incubated at 4 °C in the dark for 5–10 min. After centrifugation, the supernatant was removed, and the cells were resuspended in 200 μL of ice-pre-cooled PBS [40]. The DCFH-DA probe (Elabscience Biotechnology (Houston, TX, USA), E-BC-K138-F) was incubated in the dark at 37 °C for 60 min. After washing and removing the uninternalized probe, it was resuspended and filtered, and a positive control was set as a reference. Intracellular ROS levels were quantified by flow cytometry using an excitation wavelength of 502 nm.

#### 4.3.11. Immunofluorescence

After dewaxing and rehydration treatments, the paraffin sections were subjected to antigen retrieval. Then, they were treated with 0.1% Triton X-100 for 10 min for permeabilization, and were blocked with normal serum at room temperature for 2 h. cTnT (ab209813, Abcam, Cambridge, UK), GATA4 (307823, Abcam, Cambridge, UK) and HIF-1α (48058T, CST, Danvers, MA, USA) primary antibody (incubated overnight at 4 °C) and fluorescently labeled secondary antibody Goat Anti-Rabbit IgG H&L (ab150079, Abcam, Cambridge, UK) (incubated at room temperature for 2 h in darkness) were added. Then, the samples were washed with PBS, and the film was sealed with a DAPI-containing sealing agent. The samples were observed under a fluorescence microscope, and images were collected [41]. Quantitative analysis was performed using ImageJ software by measuring the mean fluorescence intensity (MFI).

#### 4.3.12. Immunohistochemistry

Paraffin sections were roasted at 65 °C, dewaxed with xylene and gradient ethanol to water, and then blocked with 3% H_2_O_2_ to block endogenous peroxidase. Next, the sample was rinsed with PBS solution (3 times, each for 5 min), and then antigen retrieval was performed using the heat-induced method in citrate buffer (pH 6.0). Subsequently, sections were incubated with Col I (72026T, CST, Danvers, MA, USA), Col III (68320-1-IgProteintech, Rosemont, IL, USA) primary antibody overnight at 4 °C and labeled secondary antibody Goat Anti-Rabbit IgG H&L (ab97080, Abcam, Cambridge, UK) at 37 °C for 30 min. DAB color development was visualized and controlled under a microscope. Subsequently, sections were hematoxylin-counterstained, dehydrated, cleared, and finally sealed with neutral gum. Quantitative analysis was performed using ImageJ software, and the staining intensity was assessed by measuring the average optical density (AOD) of the positively stained regions.

### 4.4. Statistical Analysis

Data analysis was performed using SPSS 20.0 statistical software. For continuous variables involving multiple groups, if the data were normally distributed and met the assumption of homogeneity of variances, one-way ANOVA was used, followed by Tukey’s test for pairwise comparisons between groups. If the data were normally distributed but variances were not homogeneous, Dunnett’s T3 test was used. If the data were not normally distributed, the Kruskal–Wallis H test was used. H&E scores were expressed as median (interquartile range, IQR). The remaining data were expressed in the form of mean ± standard deviation ($\bar{x}$ ± s). The data were plotted using GraphPad Prism 8.0 software. *p* < 0.05 was considered to be significant.

## 5. Conclusions

As a natural compound, Hyp has the advantages of multi-target and low toxicity. This study demonstrated that Hyp can substantially improve cardiac function, reduce myocardial injury and alleviate MF in ISO-induced MF mice, and high-dose Hyp (36 mg·kg^−1^) is the optimal intervention condition. This finding provides empirical support for Hyp as a potential anti-MF drug and has important transformation value. This research further confirmed that the mechanism by which Hyp improves MF may be related to the reduction in oxidative stress by activating the GATA4/HIF-1α pathway. This mechanism reveals the core position of oxidative stress in MF and provides a new target for targeted therapy. In the future, it can be further developed as a preparation or combination for adjuvant treatment of MF.

## Figures and Tables

**Table 1:** Binding affinities of Hyp with GATA4 and HIF-1α targets predicted by molecular docking.

Targets	Molecular Binding Energy (kcal·mol^−1^)
GATA4	−7.4
HIF-1α	−5.6

**Table 2:** Primer sequence.

Gene	Forward Primer	Reverse Primer
*GATA4*	AAGTCCTAGGAAACGCCCG	CATCGCGGGATGCACACAAG
*HIF-1α*	AGGATGAGTTCTGAACGTCGAAA	GGGGAAGTGGCAACTGATGA
*Col I*	GCAAGAGGCGAGAGAGGTTT	GACCACGGGCACCATCTTTA
*Col III*	ACCAAAAGGTGATGCTGGAC	GACCTCGTGCTCCAGTTAGC
*α-SMA*	GTGACTACTGCCGAGCGTG	ATAGGTGGTTTCGTGGATGC
*TGF-β*	TCAGACATTCGGGAAGCAGT	TGACGTCAAAAGACAGCCAC
*HO-1*	CCCCACCAAGTTCAAACAGCTCT	ATCACCTGCAGCTCCTCAAACAG
*cTnT*	TTCATGCCCAACTTGGTGC	CTCTCTTCAGCCAGGCGGTTC
*GAPDH*	GGCAAATTCAACGGCACAGTCAAG	TCGCTCCTGGAAGATGGTGATGG

## Data Availability

The original contributions presented in this study are included in the article. Further inquiries can be directed to the corresponding authors.

## Abbreviations

The following abbreviations are used in this manuscript:

Hyp	Hyperoside
MF	Myocardial Fibrosis
ECM	Extracellular Matrix
HFpEF	Heart Failure with Preserved Ejection Fraction
GATA4	GATA Binding Protein 4
ROS	Reactive Oxygen Species
cTnT	Cardiac Troponin T
cTn1	Cardiac Troponin I
Col I	Collagen I
Col III	Collagen III
α-SMA	α-Smooth Muscle Actin
CK-MB	Creatine Kinase Muscle-Brain
LDH1	Lactate Dehydrogenase Isoenzyme 1
LDH2	Lactate Dehydrogenase Isoenzyme 2
SOD	Superoxide Dismutase
FEL	Free-Energy Landscape
ISO	Isoproterenol
MFI	Mean Fluorescence Intensity
AOD	Average Optical Density
TGF-β	Transforming Growth Factor-β
HO-1	Heme Oxygenase-1
EF	Ejection Fraction
FS	Fractional Shortening
LVIDs	Left Ventricular Internal Dimension in Systole
LVIDd	Left Ventricular Internal Dimension in Diastole
RMSD	Root Mean Square Deviation
HIF-1α	Hypoxia-Inducible Factor-1α
EndMT	Endothelial–Mesenchymal Transition
IQR	Interquartile Range
